# TNFR2 expression by CD4 effector T cells is required to induce full-fledged experimental colitis

**DOI:** 10.1038/srep32834

**Published:** 2016-09-07

**Authors:** Xin Chen, Yingjie Nie, Haitao Xiao, Zhaoxiang Bian, Anthony J. Scarzello, Na-Young Song, Anna L. Trivett, De Yang, Joost J. Oppenheim

**Affiliations:** 1 State Key Laboratory of Quality Research in Chinese Medicine, Institute of Chinese Medical Sciences, University of Macau, Macau SAR, China; 2 Cancer and Inflammation Program, Center for Cancer Research, National Cancer Institute, Frederick, Maryland 21702, United States; 3 School of Chinese Medicine, Hong Kong Baptist University, Kowloon, Hong Kong SAR, China

## Abstract

There is now compelling evidence that TNFR2 is constitutively expressed on CD4^+^ Foxp3^+^ regulatory T cells (Tregs) and TNF-TNFR2 interaction is critical for the activation, expansion and functional stability of Tregs. However, we showed that the expression of TNFR2 was also up-regulated on CD4^+^ Foxp3^−^ effector T cells (Teffs) upon TCR stimulation. In order to define the role of TNFR2 in the pathogenic CD4 T cells, we compared the effect of transferred naïve CD4 cells from WT mice and TNFR2^−/−^ mice into Rag 1^−/−^ recipients. Transfer of TNFR2-deficient Teff cells failed to induce full-fledged colitis, unlike WT Teffs. This was due to defective proliferative expansion of TNFR2-deficient Teff cells in the lymphopenic mice, as well as their reduced capacity to express proinflammatory Th1 cytokine on a per cell basis. *In vitro*, the proliferative response of TNFR2 deficient naïve CD4 cells to anti-CD3 stimulation was markedly decreased as compared with that of WT naïve CD4 cells. The hypoproliferative response of TNFR2-deficient Teff cells to TCR stimulation was associated with an increased ratio of p100/p52, providing a mechanistic basis for our findings. Therefore, this study clearly indicates that TNFR2 is important for the proliferative expansion of pathogenic Teff cells.

Tumor necrosis factor-alpha (TNF) is a pleiotropic cytokine involved in the initiation and orchestration of inflammation and immunity^1^. Diverse biological effects of TNF are mediated by two functionally distinct receptors, namely, TNFR1 (p55) and TNFR2 (p75)^2^. Although considered a major proinflammatory cytokine, TNF also has anti-inflammatory and immunosuppressive effects^3^,^4^. We have shown that TNFR2 was constitutively expressed by Tregs and TNF-TNFR2 interaction preferentially activated and expanded naturally occurring CD4^+^ Foxp3^+^ regulatory T cells (Tregs)^5^,^6^,^7^,^8^. These surprising findings prompted other investigators to suggest that TNFR2 mediated the immunosuppressive effect of TNF, and consequently TNFR2 agonists might provide effective treatments for autoimmunity^9^. Furthermore, selective inhibition of TNFR1 was proposed to be effective in inhibiting autoimmune inflammation, and was also considered to be able to reveal the immunosuppressive effect of TNFR2^10^,^11^.

In response to TCR stimulation, mouse CD4^+^ Foxp3^−^ conventional or effector T cells (Teffs) also rapidly up-regulated their TNFR2 expression^5^,^12^. Such TNFR2-expressing Teffs were also more highly proliferative, expressed high levels of effector cytokines and were more resistant to Treg-mediated inhibition^12^. TNFR2-expressing human Teff cells induced by TCR stimulation were also reportedly maximal cytokine-producing effector cells^13^. Therefore, in addition to being critical in promoting Treg activity, TNFR2 signaling is also likely to play a role in the development of more pathogenic CD4 effector cells and consequently may contribute to the inflammatory pathogenesis of CD4 cell-mediated autoimmunity. Further clarification of this possibility would help devise more effective and safer specific TNF receptor targeting treatment.

In this study, we therefore examined the role of both TNFR1 and TNFR2 expressed by CD4 Teffs in the induction of colitis in lymphopenic Rag 1^−/−^ mice. The results showed that TNFR2, rather than TNFR1, is required for the induction of full-fledged colitis. This effect of TNFR2 was associated with the expansion as well as development of pathogenic Th1 cells in the lymphopenic mice. Therefore, in addition to its pivotal role in Tregs, TNFR2 is also critical for the function of Teffs and thus may represent a therapeutic target in CD4 T cell-mediated autoimmune inflammation.

## Methods

### Mice

Wild-type C57BL/6 mice, congenic Ly5.2 C57BL/6 mice, Rag 1^−/−^ mice, TNFR1^−/−^ mice, and TNFR2^−/−^ mice were provided by the Animal Production Area of the NCI (Frederick, MD). Frederick National Laboratory for Cancer Research is accredited by AAALAC International and follows the Public Health Service Policy for the Care and Use of Laboratory Animals. Animal care was provided in accordance with the procedures outlined in the “Guide for Care and Use of Laboratory Animals” (National Research Council; 1996; National Academy Press; Washington, D.C.). All experimental protocols were approved by NCI Animal Care and Use Committee (NCI-ACUC). Anti-mouse antibodies (Abs) purchased from BD Biosciences (San Diego, CA) consisted of anti-mouse CD3 (145-2C11), CD4 (GK1.5), CD25 (PC61), CD45, TNFR2 (TR75-89), Ki-67 (B56), INFγ (XMG1.2) and IL-17A (TC11-18H10). Leukocyte Activation Cocktail was also purchased from BD Biosciences. Functional grade purified anti-mouse CD3e (eBio500A2), CD28 (37.51) and IL-4 (11B11) Abs, Foxp3 Staining Set (FJK-16s) and anti-mouse TCRβ Ab (H57-597) were purchased from eBioscience (San Diego, CA). Functional grade anti-mouse TNFR1 (55R-170), TNFR2 (TR75-32.4) Abs and Ham IgG were purchased from Biolegend (San Diego, CA). Mouse IL-6, IL-12 and TNF were purchased from PeproTech (Rocky Hill, NJ). Human rTGFβ1 was from R&D Systems (Minneapolis, MN).

### T cell transfer model of colitis

Naive CD4^+^CD25^−^CD45RB^hi^ T cells were isolated from WT congenic B6 (CD45.1^+^) mice, or from TNFR1^−/−^ mice, or from TNFR2^−/−^ mice. The cells were injected i.p. into Rag 1^−/−^ immunodeficient recipients (4 × 10^5^ cells/mouse) separately, or cells from WT mice (4 × 10^5^ cells/mouse) were co-transferred with cells from TNFR2^−/−^ KO mice at a 1:1 ratio. Mice were monitored weekly for clinical symptoms of colitis such as rectal bleeding, loose feces/diarrhea, rough/hunched posture and loss of body weight by animal facility staff. Diseased mice were identified based on persistent clinical signs of mouse colitis such as loose feces, or diarrhea, or rectal bleeding^14^. Any mouse losing >20% of their starting body weight or showing severe signs of disease was euthanized. In some experiment, Naïve CD4 cells (CD4^+^CD25^-^CD45RB^hi^) were flow-sorted from Ly5.2 WT B6 mice (CD45.2^−^) and TNFR2^−/−^ mice (CD45.2^+^) and mixed at a 1:1 ratio. The cells (6 × 10^5^ cells/mouse) were transferred into Rag 1^−/−^ mice. The mice were sacrificed 4 weeks later and lymphoid tissues were harvest for analysis. In some experiment, MACS-purified CD4 cells from Ly5.2 WT B6 mice (CD45.1^+^) and from TNFR2^−/−^ mice (CD45.2^+^) were mixed at 1:1 ratio and i.p. injected (2 × 10^6^ cells/mouse) into Rag 1^−/−^ mice. The recipient mice were sacrificed 2 weeks later and lymphoid tissues were harvested for analysis.

### Histology

Rag 1^−/−^ recipients were euthanized 10 weeks after adoptive transfer. Colons were isolated, prepared as a “swiss roll” and were fixed in 10% formalin. Fixed tissue blocks were paraffin-embedded. 5 μm thick sections were stained with haematoxylin-eosin (HE) to assess morphological alterations and cellular infiltration. Colon sections were blindly scored according to a 5-point semiquantitiative scale described previously^15^. Images were acquired with a microdigital camera mounted on an Olympus BX40 microscope (Hicksville, NY) integrated with the software Bioquant Life Science 2015 v15.5.60 (Nashville, TN).

### Cell isolation

Single cell suspensions from spleen, mesenteric LNs (mLNs) and other regional LNs were prepared by filtration through a 70 μm cell strainer (BD Labware, San Jose, CA). Preparation of colon lamina propria (cLP) cells was as previously described^16^. Briefly, colons were rinsed in PBS and cut into ~0.3 cm pieces. Intestinal epithelial cells were removed by incubation with Ca- and Mg-free PBS containing 10% FCS and 5 mM EDTA. Colon tissues were then incubated with RPMI 1640 containing 10% FCS and 1 mg/ml collagenase type 4 (Worthington Biochemical Corporation, Lakewood, NJ) for 30 min at 37 °C. CD4^+^ T cells were purified using magnetic beads coated with anti-CD4 Ab (clone L3T4) according to the manufacturer’s instructions (Miltenyi Biotec Inc.). Subsequently, the CD4^+^ cells were stained with anti-CD4, anti-CD25, and anti-CD45RB Abs and sorted into naïve CD4^+^CD25^−^CD45RB^hi^ T cells using a Cytomation MoFlo cytometer (Fort Collins, CO, USA).

### *In vitro* T cell culture and activation

Naive CD4^+^CD25^−^CD45RB^hi^ T cells from WT C57BL/6 mice or TNFR2^−/−^ mice were seeded at 5 × 10^4^ cells/well in a 96-well plate. The cells were stimulated with plate-bound anti-CD3e Ab (10 μg/ml) alone or with soluble anti-CD28 Ab (2 μg/ml) for 3 days. In some experiments, the cells were stimulated with APCs (T-cell depleted splenic cells, irradiated at 3000 rads) and soluble anti-CD3e Ab (1 μg/ml) for 3 days. The cells were cultured in RPMI 1640 supplemented with 10% fetal bovine serum (FBS, Hyclone, Logan, UT) containing 2 mM glutamine, 100 IU/ml penicillin, and 100 μg/ml streptomycin, 10 mM HEPES, 1 mM sodium pyruvate, 0.1 mM nonessential amino acids and 50 μM 2-ME. The cell proliferation was determined by ^3^H thymidine incorporation assay or by CFSE-dilution assay.

### Flow cytometry

After blocking FcR, cells were incubated with appropriately diluted antibodies. Acquisition was performed using a SLRII (BD Biosciences, Mountain View, CA) and data analysis was conducted using FlowJo software (Tree Star Inc., Ashland, OR). For intracellular cytokines staining, cells were re-stimulated with BD Leukocyte Activation Cocktail for 4 h. FACS analysis was gated on the live cells only by using a LIVE/DEAD^®^ Fixable Dead Cell Stain Kit.

### Western blot analysis of expression of p100 and p52

Naive CD4^+^CD25^−^CD45RB^hi^ T cells were flow-sorted from WT C57BL/6 mice or TNFR2^−/−^ mice. The cell lysates (5 μg) were applied to an acrylamide gel and transferred to the PVDF membranes. The levels of protein expression were assessed by using specific antibody of p100/p52 (4882, from Cell Signaling Technology, Inc. Danvers, MA). Mouse Actin mAb (A-5441) was from Sigma (St. Louis, MO). The membranes were probed with horseradish peroxidase-conjugated secondary antibodies (Santa Cruz Biotechnology, Inc., Santa Cruz, CA).

### Statistical analysis

The cumulative incidence of colitis was graphed as a survival plot and analysed with Logrank test. A comparison of other data was analysed by Mann-Whiney U test, or two-tailed Student’s *t* test, or Two-way ANOVA test using Graphpad Prism 6.0, as indicated in figure legend.

## Results

### TNFR2 expression by Teff cells is required to induce full-fledged colitis in Rag 1^−/−^ mice

To examine the role of TNF-TNFR2 interaction in the development of pathogenic CD4 effector T cells (Teffs) in an autoimmune setting, the experimental colitis model induced by transfer of naïve CD4 T cells into lymphopenic Rag 1^−/−^ mice was utilized. In this model, a high level of TNF was expressed by both transferred CD4 Teff cells as well as by the host leukocytes present in the inflamed colon (Supplementary Fig. S1A). Although freshly isolated WT naïve CD4 cells expressed very low levels of TNFR2, this receptor was expressed by 50% of transferred CD4 Teffs present in the inflamed colon of recipient Rag 1^−/−^ mice (Supplementary Fig. S1B). Therefore, this experimental system is adequate to investigate the interaction of TNF and TNFR2 in the development of pathogenic Teff cells.

**Figure 1:**
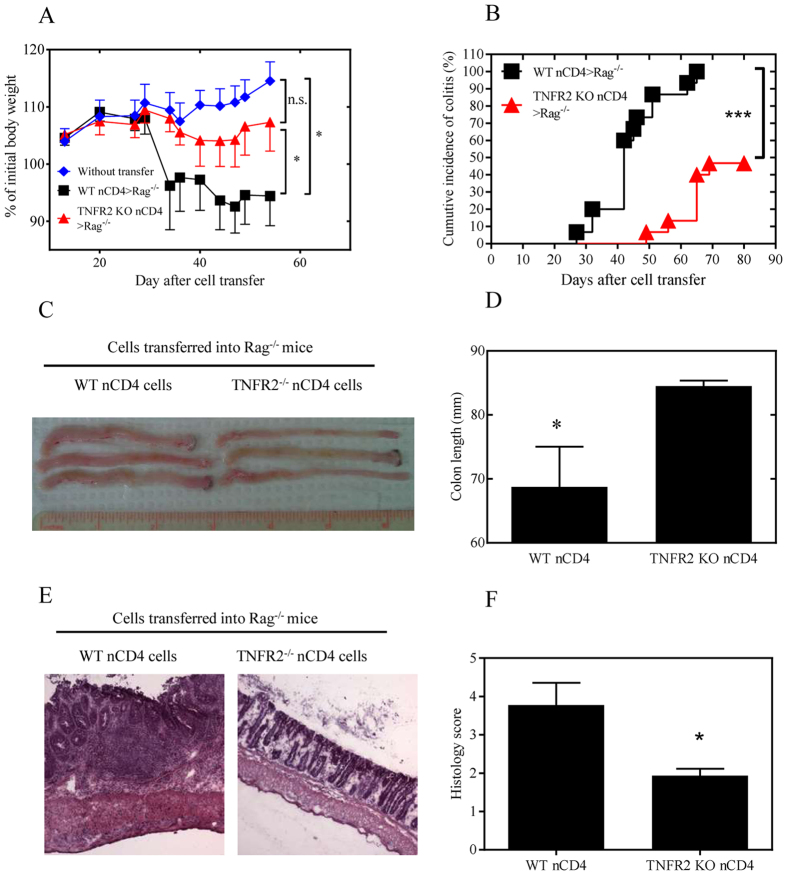
TNFR2-deficient naive CD4 cells were less colitogenic than WT cells upon transfer into Rag 1^−/−^ mice. CD4^+^CD25^−^CD45RB^hi^ naive cells from spleens of WT mice or TNFR2^−/−^ mice were transferred into Rag 1^−/−^ mice (2 × 10^5^ cells/mouse). Development of colitis was monitored. (**A**) Percent change of body weight (Means ± SEM, N = 10). Data shown are representative of at least three separate experiments with similar results. (**B**) proportion of cumulative diseased mice (%, N = 15, pooled from three separate experiments). Mice showing persistent clinical signs of colitis (loose feces, diarrhea, or rectal bleeding) were recorded as diseased mice. The differences in (A&B) were analyzed by Log-rank (Mantel-cox) test. Comparison of indicated groups, ***p < 0.0001; *p < 0.05; n.s.: not statistically different. (**C**,**D**) length of colons. Typical colon images are shown in C and summary of length of colon is shown in D (N = 4, mean + SEM). (**E**) Representative H&E-stained sections of colon from Rag 1^−/−^ recipients of WT or TNFR2^−/−^ naïve CD4 cells 10 weeks after transfer; original magnification 10 X . (**F**) Histological scoring (mean + SEM, N = 4~5). The differences in (D&F) were analyzed by Mann-Whiney test. Comparison of indicated groups, *p < 0.05. The representative data from 5 separate experiments is shown.

To compare their colitogenic effects, the same numbers of naïve CD4 cells from WT mice or from TNFR2^−/−^ mice were administered to Rag 1^−/−^ recipients. As shown in Fig. 1A, about 5 weeks after transfer, WT naïve CD4 cells were able to induce colitis in Rag 1^−/−^ mice, as indicated by a decrease in their body weight as compared with Rag 1^−/−^ mice that did not receive any transferred cells (p = 0.02). In contrast, transfer of TNFR2 deficient naïve CD4 cells failed to markedly reduce the body weight of recipient mice (p > 0.05, as compared with untreated Rag 1^−/−^ mice). Furthermore, the difference in body weight in Rag 1^−/−^ mice administered WT naïve CD4 cells compared with TNFR2^−/−^ naïve CD4 cells was significant (p < 0.05). Some of the Rag 1^−/−^ mice started to develop disease from day 27 after transfer of WT naïve CD4 cells, and all mice showed signs and symptoms of disease by day 65 (the median day to develop disease was 42, Fig. 1B). In contrast, Rag 1^−/−^ mice which were administered TNFR2-deficient naïve CD4 cells failed to show signs and symptoms of disease until ~50 days, and more than half of the mice did not show any signs of disease even by day 80 (p < 0.0001, Fig. 1B). Furthermore, the colons in Rag 1^−/−^ mice transferred with TNFR2-deficient naive CD4 T cells were markedly longer than that in mice transferred with WT naive CD4 T cells (p < 0.05, Fig. 1C,D, p < 0.05). Histological analysis revealed that transfer of WT naïve CD4 T cells induced severe colonic inflammation in Rag 1^−\−^ recipients, while transfer of TNFR2-deficient naïve CD4 T cells only resulted in mild colon inflammation (Fig. 1E,F, p < 0.05). In contrast, injection of TNFR1-deficient naïve CD4 cells into Rag 1^−/−^ mice were equally pathogenic as WT naïve CD4 cells, as indicated by the loss of body weight (Supplementary Fig. S2A). Therefore, these data clearly show that TNFR2, but not TNFR1, is required for CD4 Teff cells to induce severe colitis in lymphopenic mice.

### Deficiency of TNFR2 impairs the proliferative expansion of naïve CD4 T cells in the lymphopenic environment

**Figure 2:**
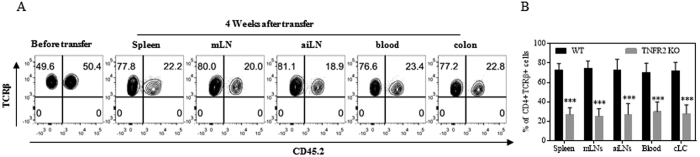
TNFR2 deficiency impairs the capacity of naive CD4 T cells to expand in the Rag 1^−/−^ mice. Naïve CD4 (nCD4) cells (CD4^+^CD25^−^CD45RB^hi^) were flow-sorted from Ly5.2 WT B6 mice (CD45.2^−^) and TNFR2^−/−^ mice (CD45.2^+^) and mixed at a 1:1 ratio. The cells (6 × 10^5^ cells/mouse) were transferred into Rag 1^−/−^ mice. After 4 weeks mice were sacrificed. (**A**) The proportion of WT CD4 T cells (CD45.2^−^) and TNFR2^−/−^ CD4 T cells (CD45.2^+^) in spleen, mesenteric LNs (mLNs), axillary/inguinal LNs (aiLNs), blood or cLP of recipient Rag 1^−/−^ mice was analyzed by FACS, gating on live CD45^+^CD4^+^TCRβ^+^ cells. (**A**) shows the typical FACS analysis and (**B**) shows the summary of data (means ± SD, N = 3). Comparison (Two-way ANOVA test) of WT and TNFR2^−/−^ CD4 cells in the same organ, ***p < 0.001. Data shown are representative of three separate experiments with similar results.

**Figure 3:**
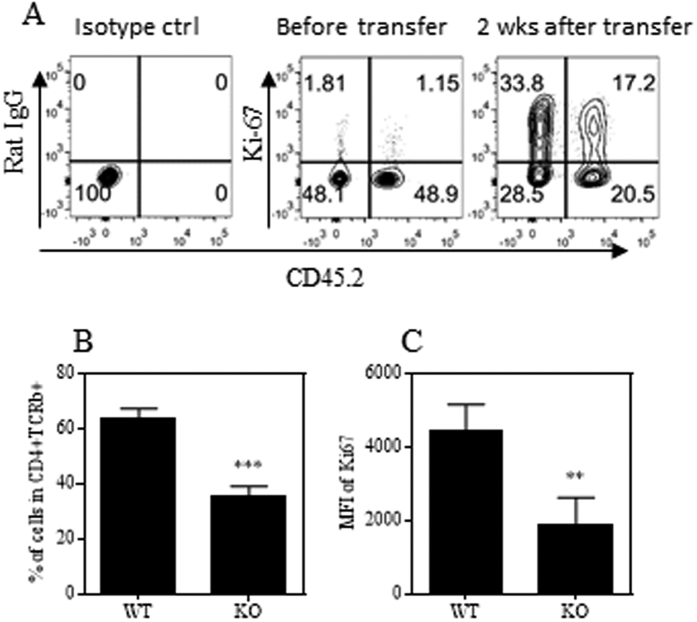
CD4 cells deficient in TNFR2 are less proliferative in Rag 1^−/−^ mice. CD4 cells were MACS-sorted from Ly5.2 WT B6 mice (CD45.2^−^) and TNFR2^−/−^ mice (CD45.2^+^) and mixed at a 1:1 ratio. The cells (2 × 10^6^ cells/mouse) were transferred into Rag 1^−/−^ mice. After 2 weeks, mice were sacrificed. The proportion of WT CD4 T cells (CD45.2^−^) and TNFR2^−/−^ CD4 T cells (CD45.2^+^), and their Ki-67 expression, in mesenteric LNs (mLNs) of recipient Rag 1^−/−^ mice was analyzed by FACS, gating on live CD45^+^CD4^+^TCRβ^+^ cells. (**A**) Shows the typical FACS analysis of Ki-67 expression by pre-transferred cells and cells recovered 2 weeks after transfer. (**B**) Shows the summary of proportion of Ki-67-expressing of WT and TNFR2^−/−^ CD4 cells (means ± SD, N = 4). (**C**) Shows the expression levels of Ki-67 on WT and TNFR2^−/−^ CD4 cells. Comparison of the difference between two groups, *p < 0.05 (N = 4, two-tailed Student *t* Test). The number in the FACS data indicates the proportion of cells in the respective gating. Data shown are representative of three separate experiments with similar results.

Upon transfer into lymphopenic Rag 1^−/−^ mice, naïve CD4 T cells were presumably activated by encountering gut microbiota and consequently underwent robust proliferative expansion. We previously reported that Tregs deficient in TNFR2 poorly proliferated in a lymphopenic environment^8^, and deficiency of IKKα, a key component of TNFR2 signaling, resulted in impaired proliferation of Tregs as well as Teff cells in Rag 1^−/−^ mice^17^. Thus it is possible that TNFR2-deficient naïve CD4 cells may also be impaired in their capacity to proliferate in Rag 1^−/−^ mice. To test this, flow-sorted naïve CD4 cells from Ly5.2 WT B6 mice (CD45.1^+^) and TNFR2^−/−^ mice (CD45.2^+^) were mixed at a 1:1 ratio and injected into Rag 1^−/−^ mice. Four weeks after transfer, the presence of WT and TNFR2-deficient CD4 cells in the blood, external LNs (inguinal and axillary region), internal LNs (mesenteric region), spleen and colon lamina propria (cLP) was examined. The results show that the ratio of TNFR2-deficient cells to WT cells was decreased from a ratio of 1:1 to a ratio of 3:1–4:1 (p < 0.0001, Fig. 2A,B). TNFR2-deficient CD4 T cells were decreased in all observed compartments, which was unlikely to be attributable to their altered pattern of trafficking or distribution. TNFR2 has been implicated to contribute to cellular survival and proliferation^18^,^19^,^20^,^21^, thus the reduction of TNFR2-deficient CD4 Teff cells may result from cell death and/or from their impaired capacity to proliferate. We observed the viability of cells activated *in vitro*, did not show any difference between WT CD4 cells and TNFR2-deficient CD4 cells upon stimulation with anti-CD3 Ab or anti-CD3 plus CD28 Abs (Supplementary Fig. S3). Furthermore, following transfer into Rag 1^−/−^ mice, the death of CD4 cells present in the external LNs, internal LNs and in the blood was assessed by Near-IR LIVE/DEAD^®^ Fixable Dead Cell Stain. The results showed no difference in the number of dead cells derived from WT mice and TNFR2^−/−^ mice (Supplementary Fig. S3B). Consequently, a greater death rate did not account for the reduction of TNFR2-deficient CD4 cells in Rag 1^−/−^ mice. Next we examined the expression of Ki-67, which is exclusively expressed by replicating cells^22^, by WT CD4 cells and TNFR2-deficient CD4 cells recovered from the spleen and mesenteric LNs of recipient Rag 1^−/−^ mice. Both the proportion of Ki-67^+^ cells as well as the expression levels (MFI) of Ki-67 on a per cell basis were markedly reduced in TNFR2-deficient cells (p < 0.01–0.001, Fig. 3A–C). Therefore, impaired proliferative expansion is responsible for the reduction of CD4 cells deficient in TNFR2 in Rag 1^−/−^ mice.

### Deficiency of TNFR2 impairs the development of Th1 pathogenic Teff cells in Rag 1^−/−^ mice

It is known that Tregs have the capacity to potently inhibit colitis in this model^8^,^16^. We therefore examined the possibility that the conversion of naïve CD4 cells into induced Tregs (iTregs) contributed to the reduction in colitis induced by TNFR2-deficient cells. Consistent with our previous report^17^, conversion of WT naïve CD4 T cells into Foxp3^+^ iTregs in Rag 1^−/−^ mice was very rare in this model. Further, there was no difference between WT and TNFR2-deficient CD4 T cells in the level of Foxp3 expression in the different lymphoid organs, including mLNs, aiLNs and blood (supplementary Fig. S4). Therefore, an increase in iTregs was not responsible for the reduction of colitis after transfer of TNFR2-deficient naïve CD4 cells.

**Figure 4:**
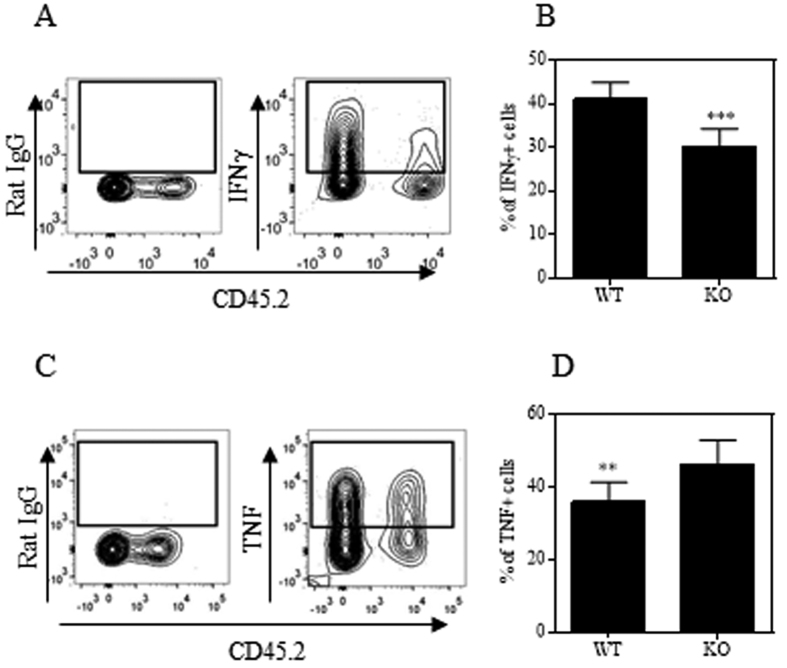
TNFR2 deficient CD4 cells express lower levels of pathogenic Th1 cytokine in the draining LNs of recipient Rag 1^−/−^ mice. Naïve CD4 (nCD4) cells (CD4^+^CD25^−^CD45RB^hi^) were flow-sorted from Ly5.2 WT B6 mice (CD45.2^−^) and TNFR2^−/−^ mice (CD45.2^+^) and mixed at a 1:1 ratio. The cells (6 × 10^5^ cells/mouse) were transferred into Rag 1^−/−^ mice. After 4 weeks, IFNγ and TNFα expressed by transferred cells recovered from mesenteric LN cells of Rag 1^−/−^ mice were analyzed by FACS. (**A**,**B**) IFNγ expression by WT and TNFR2^−/−^ CD4 cells. (**A**) Shows the typical FACS analysis, gating on live CD45^+^TCRβ^+^ cells. (**B**) Shows the summary of proportion of IFNγ-expressing WT cells and TNFR2^−/−^ cells, gating on CD45.2^−^ and CD45.2^+^ respectively of live CD45^+^TCRβ^+^ cells. (**C**,**D**) FACS analysis of TNF expression by WT and TNFR2^−/−^ cells recovered from mesenteric LNs, as described in (**A**,**B**). Comparison of two groups, **p < 0.01, ***p < 0.001 (N = 9, Two-tailed Student *t* Test). Data shown are representative of three separate experiments with similar results.

Previously we and others showed that Th1 cells were responsible for the pathogenic effect of CD4 Teff cells in this experimental colitis model^8^. We therefore examined the capacity of TNFR2-deficient CD4 Teffs to develop Th1 pathogenic cells in recipient Rag 1^−/−^ mice. As shown in Fig. 4A,B, transferred CD4 cells from both WT mice and TNFR2^−/−^ mice present in the mLNs of Rag 1^−/−^ mice had the capacity to express IFNγ. However, markedly fewer of the TNFR2-deficient CD4 cells were IFNγ-expressing cells as compared with WT cells (p < 0.0001), consistent with a previous report that TNFR2-deficient CD8 T cells expressed lower levels of this Th1 cytokine on a per cell basis^23^. The reduced IFNγ expression by TNFR2-deficient CD4 T cells was consistent detected in all organs of Rag 1^−/−^ mice, including spleen, mLN, cLPL and peripheral blood. In contrast, there were no differences in IL-17A expression by WT and TNFR2-deficient CD4 T cells in these organs (Supplementary Fig. S5A). Interestingly, more of the TNFR2-deficient CD4 cells in the mLNs expressed TNF than did the WT CD4 cells (p < 0.01, Fig. 4C,D). We next examined the capacity of TNFR2-deficient naive CD4 T cells to differentiate into Th1 and Th17 cells in the standard *in vitro* polarized culture condition. As shown in Supplementary Fig. S5B, normal levels of IFNγ could be induced in TFNR2-deficient CD4 T cells when cultured under Th1 polarized conditions. Higher levels of IL-17A expression were induced in CD4 cells deficient in TNFR2 under Th17 polarized culture condition, which was consistent with a recent study^24^. Since TNFR2-deficient CD4 T cells could be induced to express IFNγ normally *in vitro* in a standard Th1-polarized culture, the impaired expression of IFNγ by TNFR2-deficient CD4 cells in Rag 1^−/−^ mice was likely due to the lack of appropriate co-stimulation by TNF-TNFR2 interactions, rather than intrinsically impaired Th1 responses. Therefore, in addition to their impaired capacity to expand in the lymphopenic environment, reduced production of this Th1 cytokine also contributed to the reduced capacity of TNFR2-deficient naïve CD4 cells to induce colitis in lymphopenic mice.

### Deficiency of TNFR2 impairs proliferative responses of naïve CD4 cells to TCR stimulation *in vitro*

**Figure 5:**
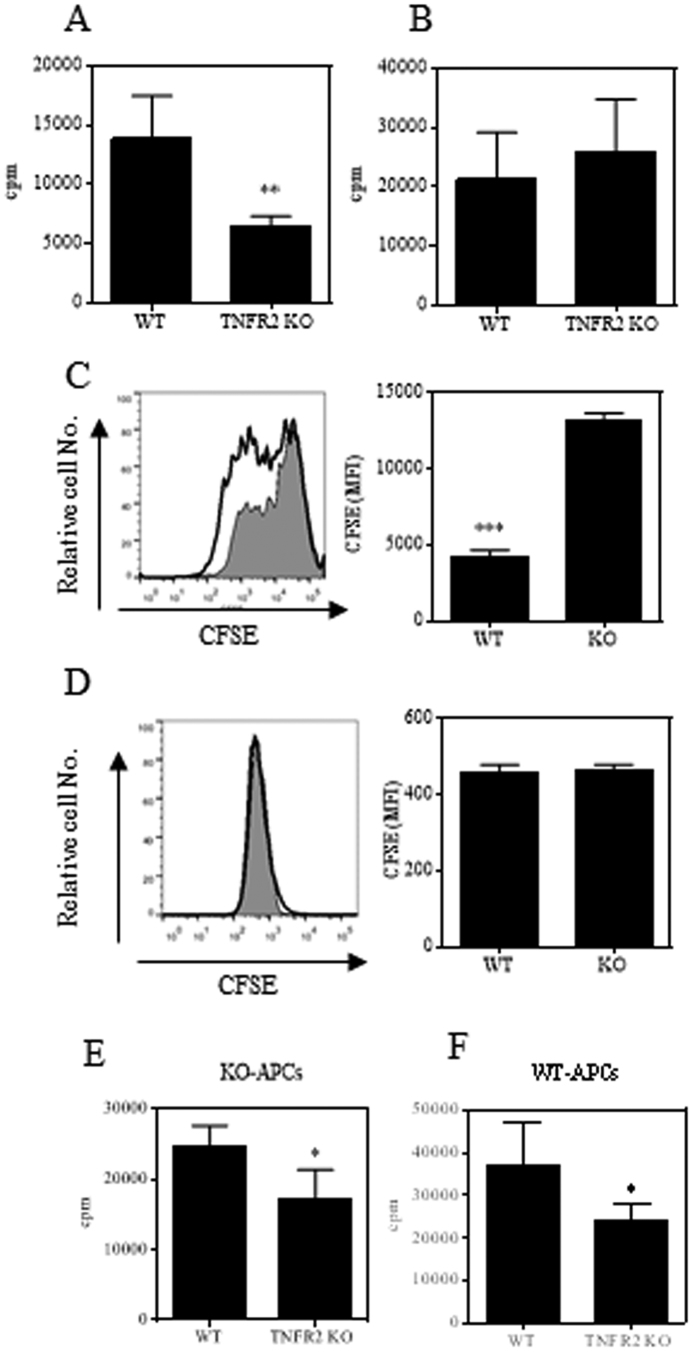
TNFR2 deficient naïve CD4 cells are less proliferative to TCR stimulation *in vitro*. Naïve CD4 cells (CD4^+^CD25^−^CD45RB^hi^) were flow-sorted from WT or TNFR2^−/−^ mice. The cells were seeded into a 96-well plate at 5 × 10^4^ cells per well. Aliquot of cells were stained with CFSE. The cells were stimulated with plate-bound anti-CD3 antibody (**A**,**C**) or with soluble anti-CD28 antibody (**B**,**D**). The proliferation of cells was evaluated either with a ^3^H thymidine incorporation assay after 72 hours incubation (**A**,**B**), or analyzed by FACS after 120 hours incubation (**C**,**D**). Grey histogram represent TNFR2^−/−^ cells, open solid line histogram represent WT cells. Typical FACS data are shown on the left and summary (N = 3, MFI of CFSE expression) was shown on the right. (**E**,**F**) WT or TNFR2^−/−^ naïve CD4 cells were stimulated with APCs either from WT mice (**E**) or from TNFR2^−/−^ mice (**F**) in the presence of 1 μg/ml of anti-CD3. The proliferation was evaluated 72 hours after incubation with a ^3^H thymidine incorporation assay. Data shown are representative of three separate experiments with similar results. Comparison of the difference between two groups, *p < 0.05, **p < 0.01, ***p < 0.001 (two-tailed Student *t* Test).

To examine if TNFR2 was required for the maximal proliferative responses of naïve CD4 cells to TCR stimulation *in vitro*, naïve CD4 cells were flow-sorted from both WT and TNFR2^−/−^ mice. Upon stimulation with plate-bound anti-CD3 Ab, WT cells proliferated robustly, while the proliferation of TNFR2-deficient naïve CD4 cells were consistently decreased by more than 50% in both ^3^H thymidine incorporation assay and CFSE labeling assay (p < 0.01~0.001, Fig. 5A,C). Nevertheless, the proliferation of TNFR2-deficient naïve CD4 cells to plate-bound anti-CD3 stimulation was restored when anti-CD28 Ab was added to the cell cultures (Fig. 5B,D), presumably the lack of TNFR2 co-stimulatory signaling was compensated for by CD28 co-stimulation. In an assay using APCs and soluble anti-CD3 as stimuli, the proliferation of TNFR2-deficient naïve CD4 cells was also markedly reduced whether APCs were from WT mice or TNFR2^−/−^ mice (p < 0.05, Fig. 5E–F). Since APCs were likely to provide multiple co-stimulants including those signaling through CD28, in this relatively more physiological condition, impaired proliferation due to the lack of TNFR2 was not compensated by the other co-stimulants in APCs.

**Figure 6:**
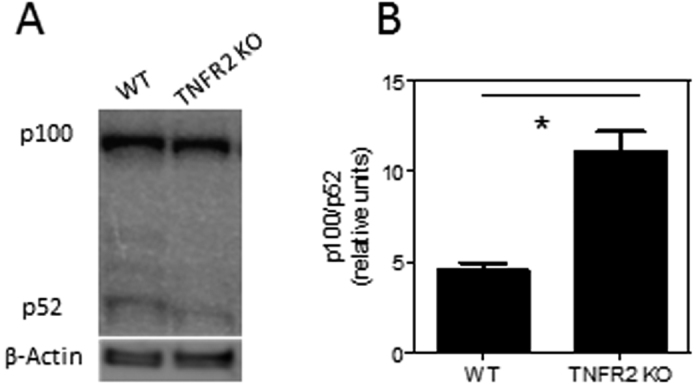
TNFR2 deficient naive CD4 T cells expressed markedly higher ratio of p100/p52. Naïve CD4 cells (CD4^+^CD25^−^CD45RB^hi^) were flow-sorted from WT or TNFR2^−/−^ mice. Immunoblotting analysis of p100 and p52 using whole cell lysates from naive CD4 T cells. Βeta-actin expression was used as loading control. (**A**) Shows a representative result of three independent experiments. (**B**) Shows the summary of ratio of p100/p52 (n = 3). Data shown are representative of three separate experiments with similar results. Comparison of the difference between two groups, *p < 0.05 (two-tailed Student *t* Test).

It has been reported that TNF-TNFR2 interaction results in the activation of non-canonical NF-κB pathway through p100 processing^25^. p100 inhibits activation of NF-κB^26^, which play a crucial role in the biological function of CD4 T cells^27^, and consequently down-regulates TCR signaling^28^. The activation of T cells is determined by the balance between p100 and p52^29^. Therefore, the expression of p100 and p52 and their ratio was examined. As shown in Fig. 6, the ratio of p100/p52 in naive CD4 T cells deficient in TNFR2 was markedly higher as compared with WT cells. Therefore, the increased ratio of p100/p52 represents at least one mechanism underlying the impaired proliferative responses of TNFR2-deficient CD4 cells.

Taken together, our data clearly show that TNFR2 expression is required for the proliferative expansion of Teff cells as well as the development of pathogenic Th1 responses in mouse model of colitis induced by transfer of naive CD4 T cells.

## Discussion

It is generally believed that TNFR1 mediates most of the proinflammatory effects of TNF^30^,^31^. In contrast, the immunosuppressive effect of TNF is thought to be mediated by TNFR2^32^,^33^, based on our discovery that TNF preferentially activated and expanded TNFR2-expressing Tregs^5^. However, recent clinical evidence suggests that TNFR2 may also play a proinflammatory role in colitis. For example, TNFR2, but not TNFR1, was up-regulated on the lamina propira and peripheral blood T cells in patients with Crohn’s disease (CD) and in the mouse colitis model^34^,^35^. TNFR2 gene polymorphisms were also associated with an increased susceptibility to Crohn’s disease^36^. Thus, TNFR2 expression may be associated with the pathogenesis of IBD. This idea is supported by our data presented in this study, which clearly indicate that TNFR2 expressed by pathogenic CD4 cells is required for the development of full-fledged colitis in a mouse model. This deleterious effect of TNFR2 on CD4 cells is associated with its crucial role in the expansion and differentiation of pathogenic Th1 cells. Our finding aligns well with the current understanding of the co-stimulatory effect of TNFR2 on T cells. For example, it has been reported that TNFR2 promotes the activation and proliferation of mouse and human CD4 T cells upon TCR stimulation^18^,^19^,^37^. In human common variable immunodeficiency patients, defective T cell activation is attributable to the impairment of the TNFR2 costimulatory pathway^38^. Furthermore, our results are most compatible with the report that transfer of TNFR2-over expressing naive CD4 cells into lymphopenic mice led to an earlier wasting syndrome, a more severe colitis and augmented Th1 cytokine production^34^. Such a co-stimulatory effect of TNFR2 was also found to be critically required for the effective priming, proliferation and recruitment of tumor–specific T cells^39^.

Binding of TNFR2 by TNF on T cells induces cIAP-1 dependent degradation of TRAF2^40^, resulting in the activation of the alternative NF-κB pathway^25^. In this process, NF-κB-inducing kinase (NIK) phosphorylates IKKα, which in turn phosphorylates p100^41^, a negative regulator of TCR signal^28^. In addition to promoting TCR responses, the activation of the alternative NF-κB pathway in T cells also enhances the production of IFNγ^42^. This can explain why TNFR2-deficient CD4 T cells expressed lower levels of pathologic IFNγ after transferring into Rag 1^−/−^ mice (Fig. 4A,B). In contrast to the decreased levels of IFNγ, TNFR2-deficient CD4 cells expressed even higher levels of TNF in Rag 1^−/−^ mice (Fig. 4C,D), which is presumably caused by the lack of negative feedback role of TNFR2 on TNF expression, as previously proposed^23^,^43^. In line with our observation, in mice with respiratory influenza virus infection, CD8 cells deficient in TNFR2 also showed a similar discrepancy in TNF and IFNγ expression^23^. To date, three signaling pathways of TNFR2 in T lymphocytes have been documented, including the IKK/NFκB, MAPK (Erk1/2, p38, JNK) and PI3K/Akt pathways^19^,^44^,^45^. In addition to IKK/NFκB, the other known signaling pathways of TNFR2 also likely contribute to the colitogenic effect of this receptor, and this possibility should be addressed in a future study.

Naive CD4 T cells deficient in TNFR2 could normally be induced to become Th1 cells under standardized *in vitro* polarized differentiation condition (Supplementary Fig. S5B), thus it is unlikely that Th1 responses are absolutely TNFR2-dependent. In addition to TCR signaling, co-stimulation is required for the priming and differentiation of T helper cells^46^. In our *in vitro* Th1 differentiation assay, the cells were stimulated with anti-CD3 as well as anti-CD28 Abs. As shown in the Fig. 5, the *in vitro* proliferative responses of TNFR2-deficient CD4 T cells to CD3 Ab stimulation can be restored by CD28 Ab. Therefore, the deficient TNFR2 co-stimulatory pathway in the *in vitro* Th1 differentiation cultures was compensated for by ligating CD28. In conjunction with the persistent impaired proliferative expansion of TNFR2 deficient CD4 T cells *in vivo* (Figs 2 and 3), TNFR2 should contribute at least in part to the *in vivo* differentiation of pathogenic Th1 cells in mouse colitis induced by transfer of naive CD4 T cells. Supportive of this idea, it was shown that the defective responses to antigens due to an impaired TNFR2 costimulatory pathway as observed in common variable immunodeficiency could not be restored by the CD28 co-stimulatory pathway^47^.

TNF purportedly plays a central role in the pathogenesis of human inflammatory bowel disease (IBD) as well as in the mouse model of colitis^48^. Anti-TNF therapy becomes indispensable in the management of IBD^49^. However, about 30% of IBD patients did not experience improvement after anti-TNF treatment^50^. This treatment also increases the risk of infections and malignancy^51^. Further, up to 10% of IBD patients receiving anti-TNF therapy developed a new autoimmune disease, including psoriasis and dermatitis-like skin reactions^52^. Therefore, dissection of the role of such TNF receptor in the colitogenic effect of TNF has obvious therapeutic implications. To this end, mouse strains with genetic ablation of TNFR1 or TNFR2 have been extensively studied in different mouse colitis models. Current studies yielded conflicting results and were thus inconclusive^53^,^54^,^55^,^56^,^57^. For example, Wang and colleagues reported that in an acute colitis model induced by instillation of dextran sulfate sodium (DSS), mice deficient in TNFR1 showed exacerbation of colitis, while deficiency of TNFR2 resulted in markedly reduced inflammation^56^. However, Punit and colleagues reported that TNFR2 deficiency resulted in a greater colitis in il10^−/−^ mice as well as in mouse colitis induced by AOM/DSS treatment^57^.

These conflicting results are likely caused by the complexity of TNF biology, different disease models and mouse strain. Furthermore, it is also possible that the role of TNFR2 in a colitis could be tissue- and cell type-specific, which has been the focus of recent studies. For example, Schneider and colleagues reported that transfer of naive CD4 T cells deficient in TNFR2 into Rag 2^−/−^ recipients resulted in an accelerated onset of disease and caused more severe colitis^21^. However, in the same mouse colitis model, e.g., transfer of naive CD4 T cell-induced colitis, the opposite result was obtained in our study. The marked differences in results may be due to different experimental condition. For example, the recipient Rag 2^−/−^ mice used in Schneider’s study^21^ were neonatally infected with *Helicobacter typhlonius* in order to accelerate the onset of colitis (Christoph Mueller, personal communication). In contrast, SPF (specific pathogen free) Rag 1^−/−^ mice were used in our study. Thus, microbiome differences may account for the opposing results.

Punit and colleagues recently reported that TNFR2 expressed by CD8 T cells ameliorate colitis by inducing death of CD8^+^ T cells^57^. The same mechanism was also reported by Schneider and colleagues in mouse colitis induced by transfer of CD4 cells^21^. Nevertheless, we failed to detect an increase in the death of CD4 cells deficient in TNFR2 in both *in vitro* as well as *in vivo* studies (supplementary Fig. S3). Actually there is evidence that TNFR2 promotes survival of T cells. For example, Kim and colleagues reported that CD4 and CD8 T cells depend on TNFR2 for survival during clonal expansion, resulting in a larger accumulation of effector cells and conferring protection from apoptosis for a robust memory pool *in vivo*^19^. They also found that TNFR2-deficient T cells exhibited defect in survival during the early phase of T cell activation that is correlated with a striking defect in Bcl-xL expression^18^. It was also reported that immunodeficient mice transferred with TNFR2-overexpressing T cells developed more severe colitis, which was associated with reduced apoptosis of T cells^34^. In patients with active Crohn’s disease, T cells were more resistant to apoptosis which was attributable to the survival signaling of TNFR2^58^,^59^. Membrane-bound TNF (mTNF) mainly binds to and activates TNFR2^60^. Biotherapeutics with the capacity to block mTNF (such as infliximab and adalimumab) have the capacity to induce apoptosis of T cells *in vivo*, and are more effective than those that only neutralize soluble TNF in the treatment of IBD^59^,^61^. Thus, both animal studies and clinical evidence do not support the claim that TNFR2 promotes T cell apoptosis in colitis.

Taken together, our data presented in this study clearly indicate that TNFR2 expressed by CD4 T cells has a deleterious effect in mouse colitis induced by transfer of naive CD4 T cells. This effect of TNFR2 is mediated by promoting the proliferative expansion of pathogenic Th1 cells. Thus, inhibition of TNFR2 or mTNF may represent a more effective and safer treatment for IBD, as compared with globally neutralizing TNF. Since Tregs express high levels of TNFR2, and the TNF-TNFR2 signaling pathway potently activates Tregs, targeting TNFR2 may impair the function of protective Tregs as a side effect. Thus, development of a strategy to selectively block TNFR2 on Teff cells would be the key to achieve an optimal therapeutic effect in IBD.

## Additional Information

**How to cite this article**: Chen, X. *et al*. TNFR2 expression by CD4 effector T cells is required to induce full-fledged experimental colitis. *Sci. Rep*. **6**, 32834; doi: 10.1038/srep32834 (2016).

## Supplementary Material

Supplementary Information
