# Corrigendum: TNFR2 expression by CD4 effector T cells is required to induce full-fledged experimental colitis

**DOI:** 10.1038/srep34680

**Published:** 2016-10-13

**Authors:** Xin Chen, Yingjie Nie, Haitao Xiao, Zhaoxiang Bian, Anthony J. Scarzello, Na-Young Song, Anna L. Trivett, De Yang, Joost J. Oppenheim

Scientific Reports 6: Article number: 3283410.1038/srep32834; published online: 09 07 2016; updated: 10 13 2016

The original version of this Article contained an error in the spelling of the author Anna L. Trivett, which was incorrectly given as Trivett L. Anna.

The Author Contributions Statement,

Conception and design, drafting of the article: X.C. and J.J.O. Performance and analysis of experiment: X.C., Y.N., H.X., A.J.S., N.Y.S. and T.L.A. Critical revision of the article for important intellectual content: B.Z.X. and D.Y.

now reads:

Conception and design, drafting of the article: X.C. and J.J.O. Performance and analysis of experiment: X.C., Y.N., H.X., A.J.S., N.Y.S. and A.L.T. Critical revision of the article for important intellectual content: B.Z.X. and D.Y.

This has now been corrected in the PDF and HTML versions of the Article.

