# Supplementary material for: TNFR2 expression by CD4 effector T cells is required to induce full-fledged experimental colitis
Source: Sci Rep. 2016 Sep 7;6:32834. doi: 10.1038/srep32834 (PMC5013387; doi:10.1038/srep32834)

Supplementary information

TNFR2 expression by CD4 effector T cells is required to induce full-fledged experimental colitis

Xin Chen[1,2*],Yingjie Nie[2], Haitao Xiao[2,3], Zhaoxiang Bian[3], Anthony J Scarzello[2], Na-Young Song[2], Trivett L. Anna[2], De Yang[2]and Joost J Oppenheim[2*]

From[1]State Key Laboratory of Quality Research in Chinese Medicine, Institute of Chinese Medical Sciences, University of Macau, Macau SAR, China;[2]Cancer Inflammation Program, Center for Cancer Research, National Cancer Institute; Frederick, Maryland 21702; [3]School of Chinese Medicine, Hong Kong Baptist University, Kowloon, Hong Kong SAR, China

Supplementary Figure legends.

Supplementary Fig S1: Expression of TNF and TNFR2 on transferred CD4 cells. Naïve CD4 (nCD4) cells ($CD4^+CD25^-CD45RB^{hi}$) were flow-sorted from WT mice or $TNFR2^{-/-}$ mice and injected into $Rag1^{-/-}$ mice (2×10^5 cells/mouse). (A) TNF expression by leukocytes in the inflamed colon. After 8 weeks of transfer of WT naive CD4 cells, single cells prepared from colon laminar propria were re-stimulated with PMA/ionomycin in the presence of GolgiPlug for 5 hours. The expression of TNF, IL-17A and IFN-γ on transferred cells ($CD4^+$) or host leukocytes ($CD4^–$) was analyzed by FACS, gating on live $CD45^+$ cells. (B) Up-regulation of TNFR2 expression on transferred CD4 T cells. Rag $1^{-/-}$ mice were transferred with WT or

$TNFR2^{-/-}$ naive CD4 T cells. Recipient mice were sacrificed 8 weeks after transfer. Expression of TNFR2 on both WT CD4 cells and $TNFR2^{-/-}$ CD4 cells was analyzed by FACS, gating on live $CD45^{+}TCR\beta^{+}CD4^{+}$ cells. Upper panel, freshly isolated naïve CD4 cells; lower panel, cells recovered from cLP of $Rag1^{-/-}$ mice 8 weeks after cell transfer. The number in the gating indicates the proportion of $TNFR2^{+}$ cells. The data shown are representatives of at three separate experiments with the same results.

Supplementary Fig S2: TNFR1-deficient naive CD4 cells were colitogenic upon transfer into Rag $1^{-/-}$ mice. $CD4^{+}CD25^{-}CD45RB^{hi}$ naive cells from spleens of WT mice or TNFR1 KO mice were transferred into $Rag1^{-/-}$ mice (2×10^{5} cells/mouse). Development of colitis was monitored. Percent change of body weight is shown. The difference was analyzed by Log-rank (Mantel-cox) test. Data are representative of two experiments with same results. N=10.

Supplementary Fig S3: Cell death of TNFR2-deficient naive CD4 cells. (A) $CD4^{+}CD25^{-}$ $CD45RB^{hi}$ naive cells from spleens of WT mice or $TNFR2^{-/-}$ mice were stimulated with plate-bound anti CD3e Ab (pCD3) or pCD3 plus pCD28 for 24 hours. The proportion of dead cells, as shown by propidium iodide (PI) staining, was analyzed by FACS. The typical FACS data are shown on the left and summary of data is shown on the right. (B) Naive CD4 cells from WT Ly5.2 mice ($CD45.1^{+}$) or $TNFR2^{-/-}$ mice ($CD45.2^{+}$) were transferred into Rag $1^{-/-}$ mice at 1:1 ratio, as described in Fig 2. Eight weeks later, mice were sacrificed and cLP were stained with LIVE/DEAD® Fixable Near-IR Dead Cell Stain Kit. The proportion of dead cells, as indicated by the Near-IR positive staining, was analyzed by FACS, gating on the $CD45^{+}TCR\beta^{+}$ transferred cells. Data are representatives of at least 3 separate experiments with same results.

Supplementary Fig S4: Expression of Foxp3 by naive CD4 cells after transfer into Rag $1^{-/-}$ mice. Naïve CD4 (nCD4) cells ($CD4^{+}CD25^{-}CD45RB^{hi}$) were flow-sorted from WT mice and $TNFR2^{-/-}$ mice. (A) The expression of Foxp3 by freshly isolated naive CD4 cells was analyzed by FACS. As a comparison, Foxp3 expression by freshly isolated $CD4^{+}CD25^{+}$ cells was shown as well. (B) WT and $TNFR2^{-/-}$ naive CD4 cells were mixed at 1:1 ratio, and injected into $Rag1^{-/-}$ mice (2×10^{5} cells/mouse). Eight weeks after cell transfer, expression of Foxp3 by both WT CD4 cells and $TNFR2^{-/-}$ CD4 cells present in the mLNs, aiLNs and blood was analyzed by FACS, gating on live $CD45^{+}TCR\beta^{+}CD4^{+}$ cells. As a comparison, proportion of $Foxp3^{+}$ cells from freshly isolated mouse CD4 T cells was shown. Data are representatives of at least 3 separate experiments with same results.

Supplementary Fig S5: IFNγ and IL-17A expression by TNFR2-deficient CD4 T cells *in vivo* and *in vitro*. (A) Naïve CD4 (nCD4) cells ($CD4^{+}CD25^{-}CD45RB^{hi}$) were flow-sorted from Ly5.2 WT B6 mice ($CD45.2^{-}$) and $TNFR2^{-/-}$ mice ($CD45.2^{+}$) and mixed at 1:1 ratio. The cells (6×10^{5} cells/mouse) were transferred into Rag $1^{-/-}$ mice. After 4 weeks, IFNγ (upper panel) and IL-17A (lower panel) expressed by transferred cells recovered from spleen, mLN, cLPL and blood of Rag $1^{-/-}$ mice were analyzed by FACS. (B) Naïve CD4 (nCD4) cells ($CD4^{+}CD25^{-}CD45RB^{hi}$) were flow-sorted from WT B6 mice ($CD45.2^{-}$) and $TNFR2^{-/-}$ mice ($CD45.2^{+}$). The cells were stimulated in the standard Th1- or Th17-polarized condition for 72 hours. Intracellular expression of IFNγ and IL-17A was determined by FACS. Comparison of indicated groups, * $p<0.05$; *** $p<0.0001$.

Supplementary Fig S1

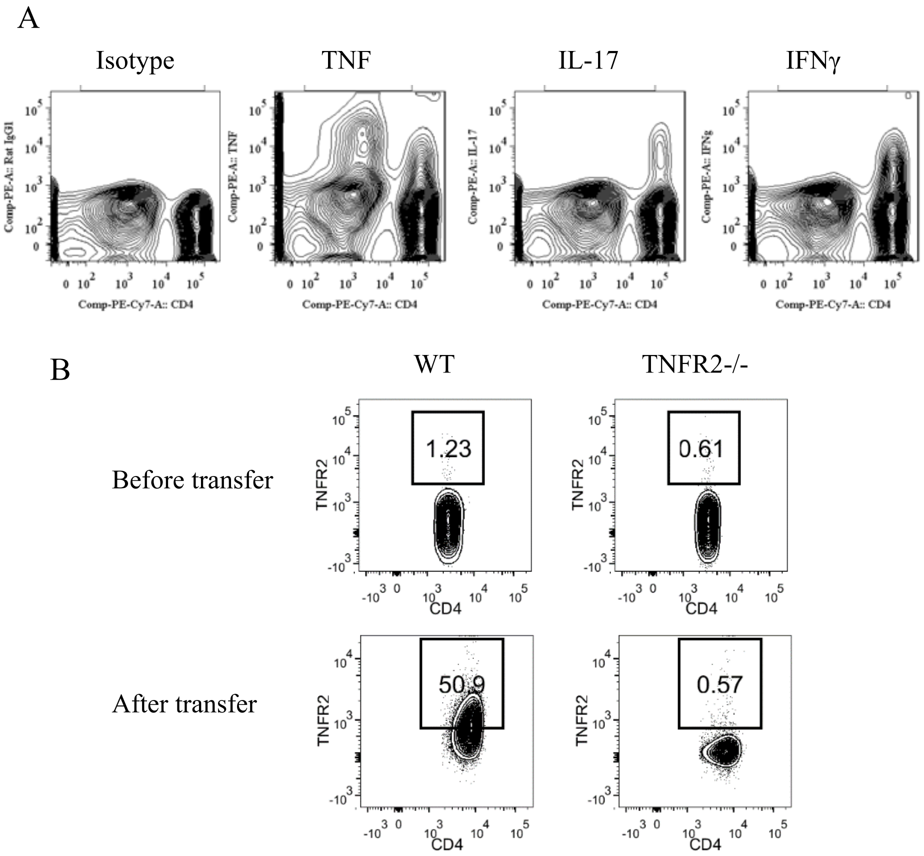

Supplementary Fig S2

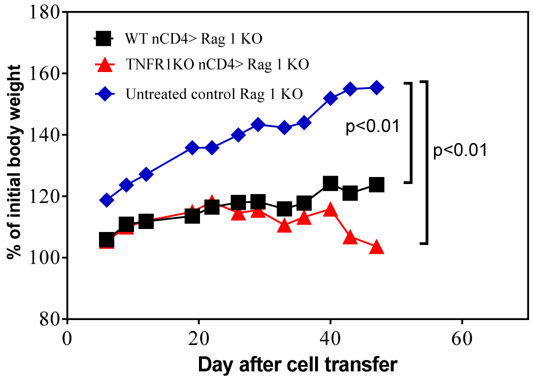

Supplemental Fig S3

A

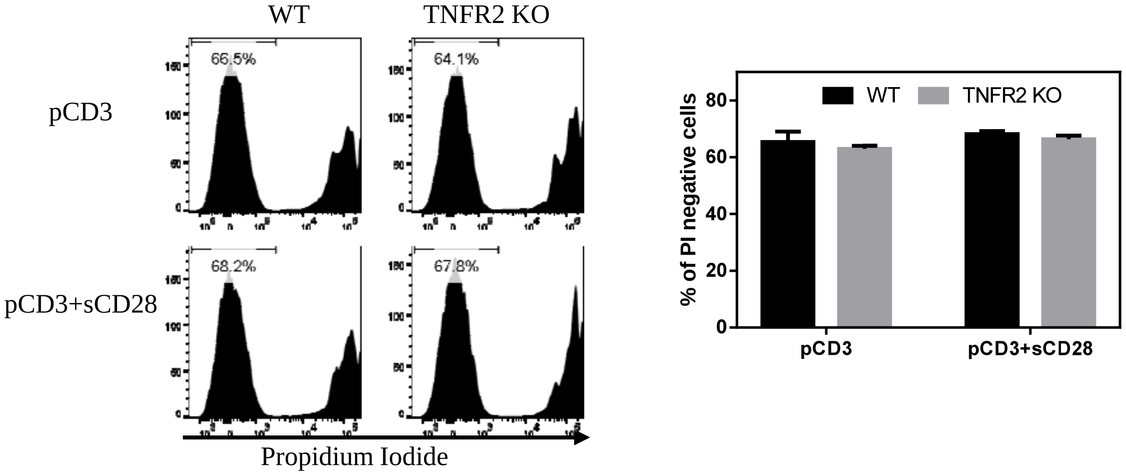

B

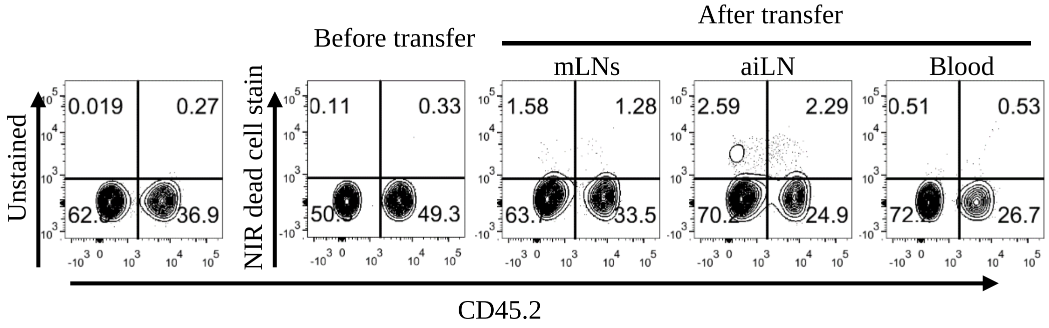

Supplementary Fig S4

A

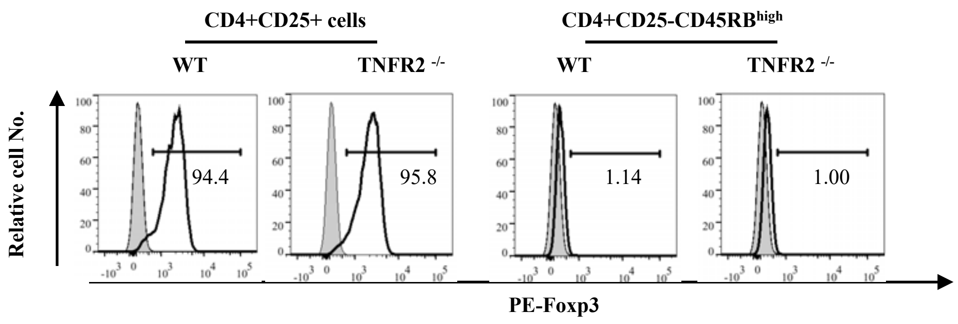

B

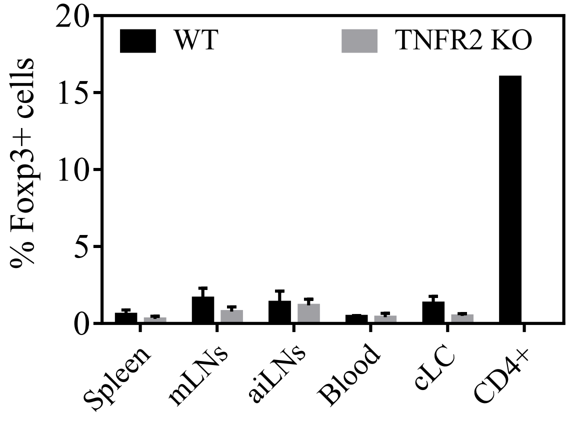

Supplementary Fig S5

A

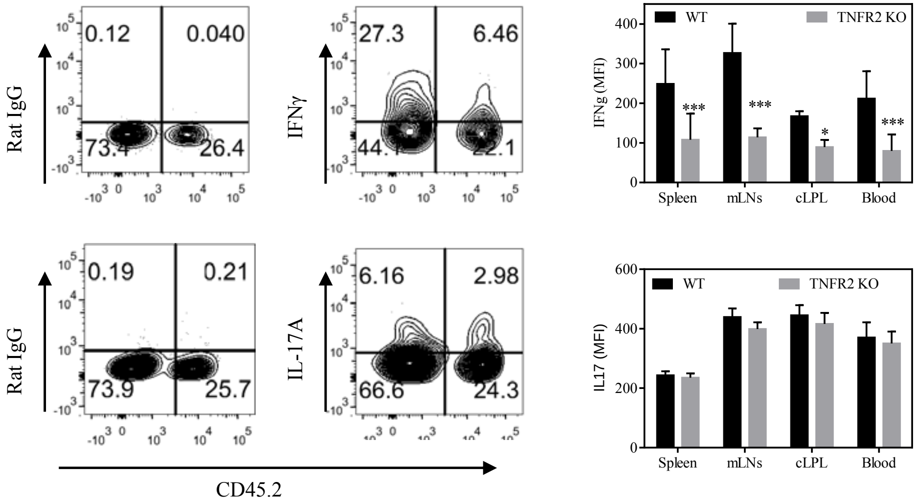

B

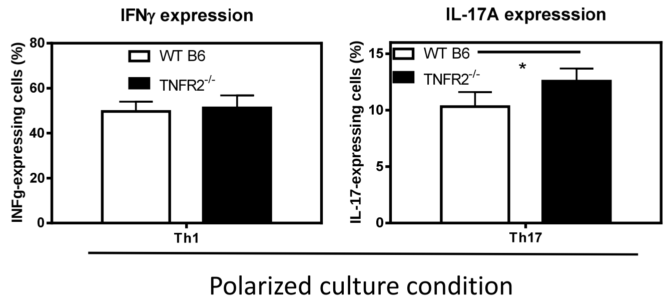

Supplement: Supplementary Information [file srep32834-s1.pdf]
